# The Adaptive Hermite Fractal Tree (AHFT): a novel surgical 3D path planning approach with curvature and heading constraints

**DOI:** 10.1007/s11548-019-01923-3

**Published:** 2019-02-21

**Authors:** Marlene Pinzi, Stefano Galvan, Ferdinando Rodriguez y Baena

**Affiliations:** Mechatronics in Medicine Laboratory, Department of Mechanical Engineering, Imperial College, London, UK

**Keywords:** Path planning, Nonholonomic constraint, Needle steering, Minimally invasive surgery, Bio-inspired, Robotic surgery

## Abstract

**Purpose:**

In the context of minimally invasive neurosurgery, steerable needles such as the one developed within the Horizon2020-funded EDEN2020 project (Frasson et al. in Proc Inst Mech Eng Part H J Eng Med 224(6):775–88, 2010. 10.1243/09544119JEIM663; Secoli and y Baena in IEEE international conference on robotics and automation, 2013) aspire to address the clinical challenge of better treatment for cancer patients. The direct, precise infusion of drugs in the proximity of a tumor has been shown to enhance its effectiveness and diffusion in the surrounding tissue (Vogelbaum and Aghi in Neuro-Oncology 17(suppl 2):ii3–ii8, 2015. 10.1093/neuonc/nou354). However, planning for an appropriate insertion trajectory for needles such as the one proposed by EDEN2020 is challenging due to factors like kinematic constraints, the presence of complex anatomical structures such as brain vessels, and constraints on the required start and target poses.

**Methods:**

We propose a new parallelizable three-dimensional (3D) path planning approach called Adaptive Hermite Fractal Tree (AHFT), which is able to generate 3D obstacle-free trajectories that satisfy curvature constraints given a specified start and target pose. The AHFT combines the Adaptive Fractal Tree algorithm’s efficiency (Liu et al. in IEEE Robot Autom Lett 1(2):601–608, 2016. 10.1109/LRA.2016.2528292) with optimized geometric Hermite (Yong and Cheng in Comput Aided Geom Des 21(3):281–301, 2004. 10.1016/j.cagd.2003.08.003) curves, which are able to handle heading constraints.

**Results:**

Simulated results demonstrate the robustness of the AHFT to perturbations of the target position and target heading. Additionally, a simulated preoperative environment, where the surgeon is able to select a desired entry pose on the patient’s skull, confirms the ability of the method to generate multiple feasible trajectories for a patient-specific case.

**Conclusions:**

The AHFT method can be adopted in any field of application where a 3D path planner with kinematic and heading constraints on both start and end poses is required.

## Introduction

Minimally invasive neurosurgery represents a major trend in modern neurosurgery, as it can minimize patient trauma, and thus the risk of complications and recovery time. Steerable needles are a promising technology in this medical field because of their ability to reach a target following a three-dimensional (3D) curvilinear trajectory, which can avoid anatomical brain structures such as blood vessels and eloquent areas. These could provide a step change in the delivery of diagnostic sensors and therapies to the brain via flexible surgical access, with the potential for advancements in cancer therapy. In this context, the EDEN2020 project, which is supported by the European Commission Horizon 2020 programme (www.eden2020.eu), is developing a biologically inspired, nonholonomic steerable needle [15, 19], for application to Convection Enhanced drug Delivery (CED, [20, 23]). While the clinical efficacy of the system has yet to be established, full 3D steering without spin [2, 13, 16] during insertion, has already been demonstrated under laboratory conditions. The underlying approach is particularly advantageous in neurosurgery owing to the delicate nature of brain tissue. EDEN2020 features a nonholonomic “programmable bevel-tip” (PBN) steerable needle that has a finite orientation velocity and an insertion mechanism inspired by the ovipositor of the wood wasp [9]. Nonholonomic robots, similar to car-like robots and fixed-wing unmanned aerial vehicles (UAVs), are incapable of performing stationary turns, as their turning radius is bounded. As a consequence of these kinematic constraints, feasible paths that the needle can track must be smooth and have bounded curvature derivatives.

Path planning algorithms are a major topic of study in the robotics literature. Their aim is to generate feasible paths from a starting point to a target point, which avoids obstacles in the search space. However, the main issue is not the generation of an obstacle-free trajectory, but the need of some systems for smoothness and curvature gradients which can be limited depending on the application.

Several methods already exist for the path planning of mobile robots, which have been or could potentially be extended to steerable needles. A promising method in the literature uses Dubins curves, where circular arcs and straight lines are combined to generate feasible paths between configurations. A study by Hota and Ghose [10] focuses on determining the optimal path for a constant-speed and turn-rate constrained vehicle, such as a fixed-wing UAV through 3D Dubins curves, while Cai et al. [4] investigates the task assignment and path planning problem for multiple UAVs in a 3D environment. Promising results have also been published by Elbanhawi and Simic [7] using a two-stage path planner based on a variation of the classic rapidly-exploring random tree (RRT) algorithm [12], which accounts both for maximum curvature, heading constraints and real-time performance in a simple geometric two-dimensional (2D) obstacle map. These methods, however, have some limitations when the maximum curvature value and the search space are very restricted; therefore, they were found not to be suitable for the application scenario behind our current neurosurgical study.

Concerning minimally invasive surgery applications, Alterovitz et al. [1, 6] proposed a method which results in a trajectory that avoids obstacles while accounting for needle motion uncertainties. Alterovitz et al. [17] also presented a rapidly-exploring road map-based approach for path planning. However, it does not guarantee accuracy on the requested target pose. Caborni et al. [3] proposed a method for the EDEN2020 catheter using RRTs and a risk-based cost function to select the preferable path; however, the problem is simplified to a 2D scenario and the target heading is still not considered as a constraint. Fause et al. [8] proposed two RRT-based techniques which make use of multiple trees departing from both start and target poses. The generated paths meet both heading and kinematic constraints for a ray of curvature equal to 2 mm. The suggested “B-RRT-Connect” makes use of 3D Dubins curves to link the two trees smoothly but, as described in [10], this method is applicable only to “points which are far enough” with respect to the curvature constraint, in particular, the distance between them has to be greater than four times the maximum acceptable curvature ray. The second proposed method is the “SB-RRT-Connect” which is based on splines and uses the Yang and Sukkarieh [21] Bézier-based algorithm to link the two RRTs satisfying an upper bounded curvature constraint. However, this smoothing algorithm struggles to find a solution when the ray constraint is equal to 70 mm (such as for our PBN), especially when the path control points are really close to each other.

Additionally, the computation time for RRT-based algorithms is dependent on the environment, as well as on the randomness of the search. These do not meet the “single instruction multiple data operations” Graphics Processing Unit (GPU) requirement; therefore, they are only scalable to multiple CPUs, with a limited speed improvement [11]. AFTs, on the other hand, run on the GPU, providing a higher success rate in real time, regardless of the number and complexity of the obstacles. As demonstrated in [14], an RRT finds a solution in a complex surgical environment in 42$\%$ less cases than an AFT due to its implementation which exploits GPU power to generate a high number of solutions in parallel. The obstacle collision check is voxel-based, the obstacle map is generated from segmentation of the patient’s magnetic resonance imaging (MRI) of brain arteries and ventricles and every branch of the tree is checked for collision in parallel; if a collision occurs, the branch is removed from the tree before computing the possible solutions. This improves the use of computational resources even further.

The complexity of the obstacle map, together with the constraints on the maximum path curvature (caused by mechanical limits of the flexible probe [13, 15]) and the requested starting and final headings, contributes to make path generation within the EDEN2020 context particularly challenging. The Adaptive Hermite Fractal Tree (AHFT) approach described in this work represents our latest attempt to address these issues. The AHFT is a hybrid approach based on the union of an optimized Adaptive Fractal Tree (AFT) algorithm with a so-called Hermite Extension method based on optimized geometric Hermite curves (OGH) [22]. The AFT already runs in parallel on the GPU, which enables fast sampling of the obstacle-free search space between the start and target points as previously mentioned. The parallel structure of the algorithm proposed here lends itself to a similar level of parallelization, which would be a necessary condition for eventual intraoperative deployment, although speed optimization is not the subject of the current publication.

The paper is structured as follows. First, an AFT automatic parameter tuning process is implemented in “AFT automatic parametrization” section, with the aim to enhance AFT performance. Then, the AHFT hybrid approach is described in “AHFT algorithm implementation” section and tested through simulated trials in “AHFT robustness evaluation” section, with a segmented human volumetric dataset use as an example.

## Background

**Fig. 1:**
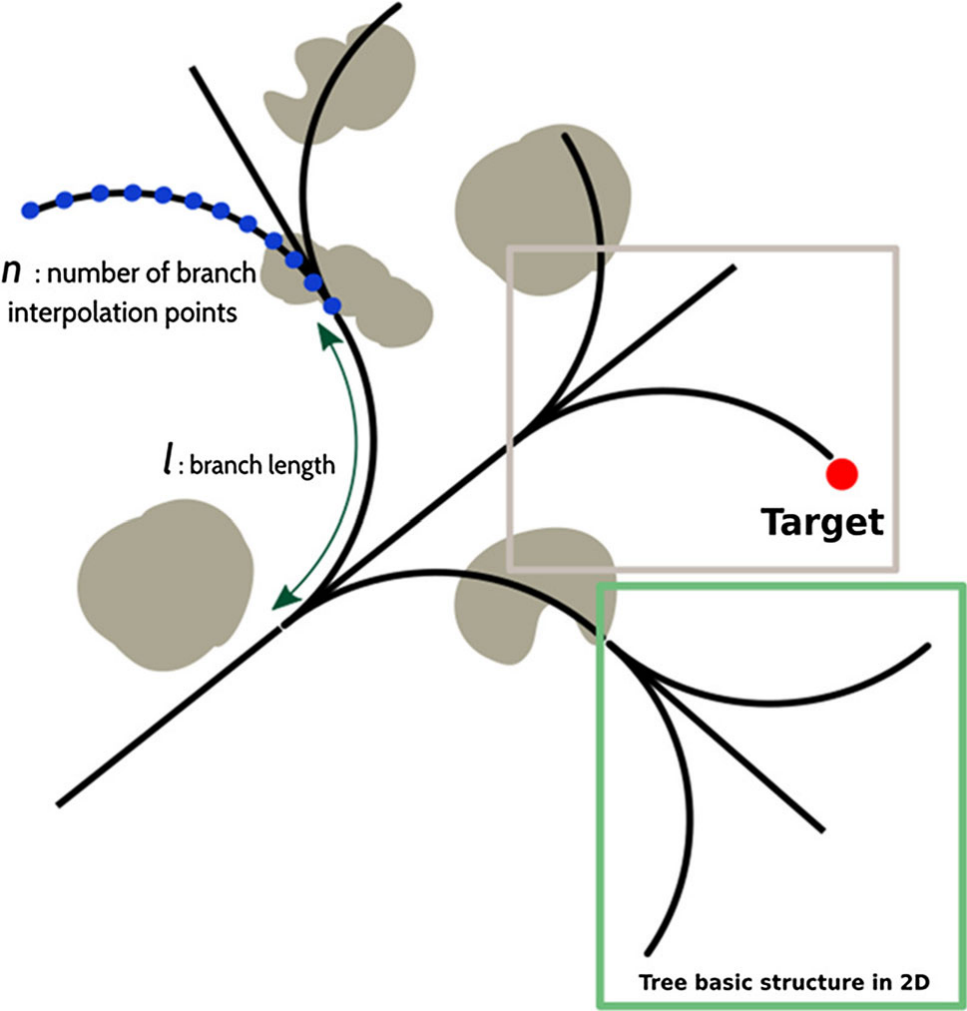
Geometry of the tree in 2D with a focus on the basic structure of the fractal geometry (within the green window)

The AFT is an algorithm built on the recursive generation of a self-similar (fractal) structure within the search space. Each branch of the tree is associated with a GPU thread and runs in parallel on the graphics card, thus optimizing the use of computational resources. The topological structure of the tree resembles the recursive nature associated with the motion of nonholonomic needles. At each step, all possible future motions depend on the current pose, a process that reverses recursively from the target to the insertion point via all tree levels. The basic tree structure with this fractal geometry features five possible motion directions: straight, up, down, right and left, represented by a straight line in the first case and arcs for all remaining cases, the curvature value of which can be chosen based on the needle’s constraints (Fig. 1). The search space between the straight line and the maximum curvature arcs can be explored by adding further arcs featuring lower curvature. For instance, if there are two arcs for each direction, the amount of possible motion directions, which is labeled as the tree density parameter ($\rho$), increases from five to nine. The trees basic structure can be shaped according to any given scenario by tuning a set of fundamental parameters associated with the trajectory generation and the needle design parameters. These include the branch length *l* and the density of the tree $\rho$.

However, the AFT is not able to deal with both starting and final heading constraints, which are required in complex neurological planning such as the one proposed in EDEN2020, where both the pose of the entry key-hole in the skull and the pose of the drug delivery target site must be defined *a priori*. Specifically, while the direction of growth of the tree can be changed according to the required starting heading, the final heading is not taken into account during path planning.

Following these considerations, the Adaptive Hermite Fractal Tree (AHFT) path planner is proposed in this work. Thanks to some important properties related to a particular kind of Hermite curves called optimized geometric Hermite curves (OGH), the AHFT is able to account for both heading constraints accurately. Given two endpoints and two endpoint tangent vectors, a cubic polynomial curve is called an OGH curve with respect to the given endpoint conditions if it has the smallest strain energy among all cubic Hermite curves satisfying the conditions on starting and final pose and is also geometrically smooth [22]. This specific property allows us to use OGH curves to extend the AFT obstacle-free paths at different positions along candidate trajectories. For each linking position, the local tangent is computed, which is subsequently employed as the starting heading for the Hermit curve connecting the AFT path to the desired target pose. Finally, a voxel-based obstacle collision and a maximum curvature check are performed on the Hermite extension with the same voxel-based modality of the AFT but evaluating one path at the time. Only feasible paths are returned.

**Fig. 2:**
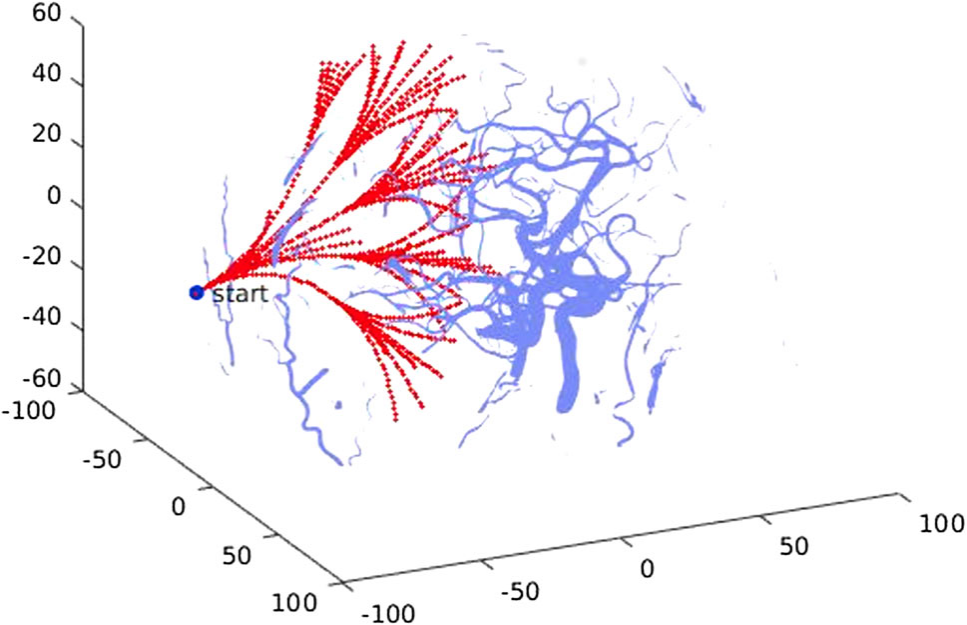
Adaptive fractal tree (AFT) extending through segmented brain vessels which constitute to the obstacle map

## Methods

### AFT automatic parametrization

First, an automatic parameter tuning process for our AFT implementation is designed to maximize the number of paths generated in a neurosurgical scenario (Fig. 2) and to exploit the entire GPU memory available during fractal tree parallel construction. To do so, it is necessary to understand the relationship between the parameters *l* (branch length) and $\rho$ (tree density) on AFT performance, an objective that we pursue through a brute force search of the parameter space.

The AFT density relates to the total number of segments composing the tree which, if beyond a threshold (number of GPU cores * 10, and equal to 25,600 for our workstation), can cause the GPU to have to iterate in order for all the segments to be processed, thus increasing computation time. On the other hand, *l* affects the number of subsegments composing each branch through which the obstacle collision has to be performed. This part of the code runs in parallel for every segment and influences the total amount of simulation time. In our simulations, the interpolation constant determines the resolution of the obstacle collision check performed for each tree branch. In order to set its value, blood vessels are segmented from a representative and anonymized MRI image volume (from EDEN2020’s private repository) via standard thresholding (www.slicer.org). Next, the branch length *l* is assigned to one of the following arbitrary values: 20, 30, 40, and 50 mm. This set is chosen in order to measure the AFTs behavior across a representative range. In particular, the minimum branch length of 20 mm comes as a result of two existing constraints, one on the maximum reachable needle length of 100 mm and the other one on the required GPU memory space which limits the tree growth to a maximum of 5 levels of increments of the fractal structure, with a density value of up to 17. Therefore, the lower limit of 20 mm has been selected as the minimum acceptable branch length value in order to cover a distance of at least 100 mm in five steps of tree growth. On the other hand, the upper limit, which cannot be more than the total path length, has been set to 50 mm in order to cover the search space with the minimum number of 2 increments. Branch lengths of 30 mm and 40 mm are chosen to assess the performance in the case of 4 and 3 increment levels, respectively. Knowing the specified branch length and the corresponding necessary levels of tree growth to cover the maximum needle extension, we can then use the exponential law governing tree growth in order to compute the total number of AFT branches needed, each of which occupies a thread of the GPU memory (Eq. 1).

1 $$\begin{aligned} \hbox {AFT}_\mathrm{branches} = \sum _{i=0}^{\textit{tree}_{\text {levels}}} ({\hbox {branch}_\mathrm{density}})^{i} \end{aligned}$$

Ultimately, the density of the AFT is set to one of these values: 9, 17 or 33. These define the number of branches forming the basic fractal structure, which has to be equal to a multiple of 4 (up, down, right, left) plus 1 (straight). A high tree density increases the mapping capability of the AFT, but this value must be limited to 33 in order to meet the GPU memory constraints, at least with the longest branch selection of 50 mm. Of particular importance is understanding the trade-off relationship between $\rho$ and *l* in terms of computational cost and AFT outcome. We explore this via a brute force search of the parameter space, where the initial tree space orientation is also considered as variable. The tree can be oriented toward random points inside a circumference around the target, which lies on the plane perpendicular to the line subtended between insertion and target points. This further variability increases the number of cases explored.

We executed a total of 1260 simulations on a workstation with an NVIDIA GeForce GTX 1080 Ti 11GB Pascal, varying parameter values as discussed above. Each path is evaluated by the cost function (CF):

2 $$\begin{aligned} \hbox {CF}= & {} w_1 * \sum _{i}^{n_\mathrm{seg} -1} \frac{|C_{i+1} - C_{i}|}{2*\max _\mathrm{curv}} +w_2 * \sum _{i}^{n_\mathrm{seg}} \frac{|C_i|}{\max _\mathrm{curv}}\nonumber \\&+\, w_3 * \frac{\hbox {length}}{\max _\mathrm{length}} \end{aligned}$$

where $n_\mathrm{seg}$ represents the number of segments composing the path. The cost function considers the preferential path characteristics, where the first term of the equation is the gradient of curvature which is calculated as the sum of the difference of curvatures ($C_{i+1} - C_i$) between consecutive branches constituting the path with respect to the maximum achievable value measured in proximity of an inflection point between two arcs of maximum curvature ($\max _\mathrm{curv}= \frac{1}{70}$) and opposite sign. It measures the smoothness of the trajectory in terms of the absence of inflection points and curvature variability. This feature is preferred because it facilitates the control of the needle movement. The second term refers to the sum of the segments’ curvature *k* with respect to the maximum curvature value, which reflects the linearity of the path. Trajectories presenting moderate curvature, away from the maximum constraint of the needle, are favored. The path length, which is normalized with respect to the maximum path length ($\max _\mathrm{length}= {tree_levels} * l$), is minimized to favor short paths, with the effect of reducing potential tissue damage during needle insertion. The three function terms are weighted by their corresponding coefficient, where $w_1=w_2=w_3=1$ in order to give all the same contribution. For each simulation, from the entire set of collision-free generated paths, the optimal path is the one which minimizes the cost function value.

### AHFT algorithm implementation

The AHFT is implemented with two main routines. The first identifies whether a given start-end pose combination is feasible. The second computes the optimum continuous path between the two, which meets the needle’s constraints and is obstacle free.

*Step I: Start and Target Pose Reachability Check* A feasibility check is performed once the path planner is invoked as a means to ascertain whether the start and target pose combination is feasible either in terms of maximum needle length or in terms of needle kinematic constraints, such as the maximum curvature constraint ($k_{\max }$). We define a “reachable volume” by considering the intersection of the two bounding volumes which enclose all branches of the two AFTs, the first rooted at the start pose and the second at the target pose. If no intersecting volume which encompasses both poses is available, the algorithm cannot progress.

*Step II: Optimized Geometric Hermite (OGH) Extension* Before defining an Optimized Geometric Hermite (OGH) curve it is appropriate to introduce the wider class of Hermite curves. The following paragraph refers to the results presented in [22], which are of particular interest for our application. The verbatim-copied parts are enclosed in quotation marks.

“A cubic Hermite curve *Q*(*t*), $t\in [t_0,t_1]$ where $(t_0,t_1)\in R$ and $t_{0} < t_{1}$ , is a cubic polynomial curve satisfying the following endpoint location and tangent vector conditions: ”

3 $$\begin{aligned} Q(t_{0})=P_{0}, Q(t_{1})=P_{1}, \overline{\mathrm{Q}}(t_{0})=V_{0}\overline{\mathrm{Q}}(t_{1})=V_{1} \end{aligned}$$

where $P_{0}$ and $P_{1}$ are the start and end point coordinates, and $V_{0}$ and $V_{1}$ represent the desired approach direction for $P_{0}$ and $P_{1}$, respectively. *Q*(*t*) can be expressed as follows:

4 $$\begin{aligned} Q(t)= & {} (2s +1){(s -1)}^{2}*P_{0}+(-2s +3){s}^{2}P_{1}\nonumber \\&+(1-s)^{2}s(t_{1}-t_{0})V_{0}+(s-1)s^{2}(t_{1}-t_{0})V_{1} \end{aligned}$$

where $$s = \frac{t-t_{0}}{t_{1}-t_{0}}$$.

“The strain energy of a $C^{2}$-continuous curve *f*(*t*) defined on [*t*0, *t*1] is defined as follows:

5 $$\begin{aligned} \int _{t_{0}}^{t_{1}} {{[f(t)'']}^{2}\hbox {d}t} \end{aligned}$$

where $[f(t)'']$ is the second derivative of *f*(*t*). A Hermite curve is mathematically smooth because it has the minimum strain energy among all $C^{1}$ cubic polynomial spline curves satisfying the same endpoint conditions”.

Between similar Hermite curves, we select the one with the minimum strain energy which is mathematically smooth by optimizing the magnitudes of the considered endpoint tangent vectors. This curve is defined in [22] as an optimized geometric Hermite (OGH) curve.

Given two endpoints $P_{0}$ and $P_{1}$, and two endpoint approach vectors $V_{0}$ and $V_{1}$, an OGH curve is the cubic Hermite curve *Q*(*t*), $t\in [t_0,t_1]$, with the smallest strain energy, and which also satisfies the following conditions:

6 $$\begin{aligned} Q(t_{0})=P_{0}, Q(t_{1})=P_{1}, \overline{{Q}}(t_{0})=a_{0}V_{0}, \overline{{Q}}(t_{1})=a_{1}V_{1} \end{aligned}$$

where $a_{0}$ and $a_{1}$ are arbitrary real numbers that relate to the magnitude of the curve approach vector in $P_{0}$ and $P_{1}$, respectively. In this study, we consider $a_{0}=a_{1}=a*$ to enforce similar conditions for both start and end pose alignment. In some cases, one might want to hold the ratio of the tangent vector magnitudes unchanged in order to maintain a fixed shape style on the resulting curve, avoiding higher curvature values at one proximity. Therefore, we give $a*$ the value suggested in [22].

7 $$\begin{aligned} a_{0}=a_{1}=a*= \frac{3[(P_{1}-P_{0})(V_{0}+V_{1})]}{2(V_{0}^{2}+V_{0}V_{1}+V_{1}^{2})(t_{1}-t_{0})} \end{aligned}$$

**Fig. 3:**
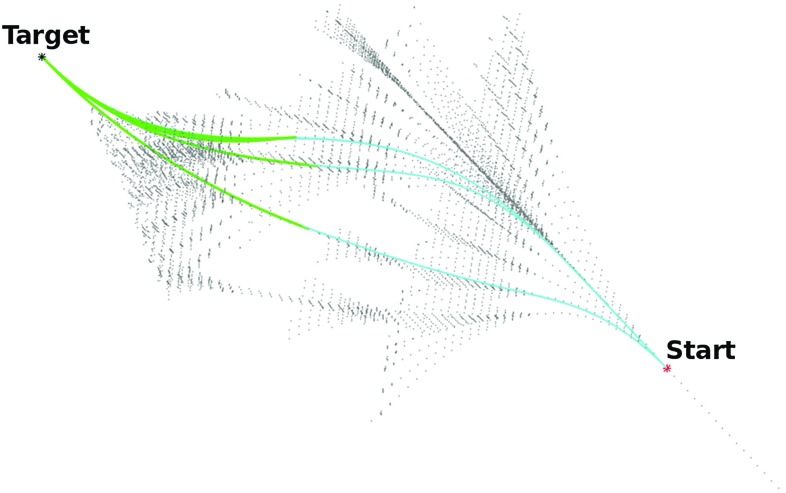
Three feasible paths, generated by the AHFT path planner, connect the requested start and target pose. In gray: the AFT tree branches laying between the feasible volume; in light blue: the AFT portion of the paths; in green: the OGH portion of the paths

In the AHFT, OGH curves are used as extensions which depart from the AFT obstacle-free paths at different positions along the trajectories and connect them to the target point with a predefined orientation (Fig. 3). First, the optimized AFT algorithm described in “AFT automatic parametrization” section is used as a method to explore the search space. Only the branches which do not collide with obstacles and which lie within the “reachable volume” identified in Step I are considered, and candidate paths are computed as per the original AFT algorithm. At this point, every point of all trajectories found so far, is considered as the initial point during the AFT extension through OGH curves, which is described next. The OGH approach vectors for all candidate extension paths are chosen such that each pair is tangent to the tree path on one side, and the desired target pose on the other. Subsequently, the generated OGHs can be easily expressed as a Bézier curves with four control points. The cubic polynomials, expressed in Bézier form, allow for an easier computation of the curvature values along the paths. Once a voxel-based collision check and a curvature checks are performed also on the candidate extensions, viable AHFT paths are considered as possible solutions and the cost function (Eq. 2) is employed to rank these in order of performance. Finally, we use a function called interparc (author: John D’Errico [5]), available on the MATLAB (Mathworks Inc.) file exchange server in order to obtain equally spaced points along the considered paths.

## Results

### AFT performance parameters significance

As already discussed in “AFT automatic parametrization” section, we executed a total of 1260 simulations, 105 for each combination of parameters which can vary as shown in table (Table 1). In particular, the AFT density $\rho$ can take the following values: 9, 17 or 33, while the AFT branch length *l* can be equal to 20, 30, 40 or 50 mm, values which are associated with the following tree expansion numbers, respectively: 5, 4, 3, 2. In this specific scenario, start and target points are chosen to be at the maximum acceptable distance with respect to the needle length. Additionally, the AFT algorithm has been iteratively run rotating the tree around an axis corresponding to its root (the entry pose) for ten times in *pi*/5$^{\circ }$ steps in order to achieve a more homogeneous and dense expansion through the search space.

These first results show that the branch length or, more generally, the related number of tree increments, have a particularly strong influence on the memory requirements. Indeed, they directly affect the number of segments of the tree and, therefore, the number of generated paths (Fig. 1). Setting $l =$ 20 mm, the maximum acceptable value of $\rho$ within the memory constraint is equal to 17. This combination produces at least one AFT path 94.23$\%$ of the times, with an average computation time of 0.22 s and an average number of solutions equal to 408.51; while, increasing the branch length, the percentage success rate decreases to 72.12$\%$ for *l* = 30 mm (4 levels of growth) and then drops to 36.54$\%$ for *l* = 40 mm (3 levels of growth) and to 25.00 $\%$ for *l* = 50 mm (only 2 levels of growth). For smaller values of AFT density, as for $\rho$ = 9, the performance of the AFT is still high, with a 90.48 $\%$ success rate. The average number of paths found is only equal to 32.82, but this is in favor of a lower computation time of 10.65 ms. Because we are interested in maximizing the AFT’s mapping ability in a complex environment, the number of generated paths together with the percentage success rate is considered as the most important indexes assessing the robustness of the method in finding solutions. Therefore, 5 levels of tree increments and $\rho$ = 17 are selected as the best combination for future trials. The computation time in a preoperative scenario is secondary with respect to the quality and robustness of the planning. In addition, the difference in computation time for *l* = 20 mm between $\rho$ = 17 and $\rho$ = 9 is due to GPU overflow, as described earlier. This unwanted side effect of an excessive number of segments would disappear if using a multi-GPU implementation or a different GPU featuring a higher number of threads was employed. In other words, an increase in $\rho$ would not affect the computation time for one GPU thread or the computation time for any given iteration, as long as the GPU features a sufficient number of threads to service all tree branches in one run.

**Table 1:** Success rate, average number of paths found, average computation time and total number of paths found for 150 trials, for each combination of length (*l*), and density ($\rho$)

Performance	Density		
	9	17	33
Length: 20 mm (5 levels)			
Success rate	90.48%	94.23%	Out
Avg *n* path	32.82	408.51	Out
Avg time	10.65 ms	223.28 ms	Out
Tot paths	3118	40,034	Out
Length: 30 mm (4 levels)			
Success rate	40.00%	72.12%	86.40%
Avg n path	5.26	18.27	117.77
Avg time	2.79 ms	18.41 ms	247.8 ms
Tot paths	221	1370	10,640
Length: 40 mm (3 levels)			
Success rate	20.95%	36.54%	53.39%
Avg n path	1	3.71	8.47
Avg time	1.41 ms	2.85 ms	10.76 ms
Tot paths	22	141	481
Length: 50 mm (2 levels)			
Success rate	20.95%	25.00%	34.95%
Avg n path	1	1.04	10.89
Avg time	1.08 ms	1.32 ms	3.05 ms
Tot paths	22	27	403

**Fig. 4:**
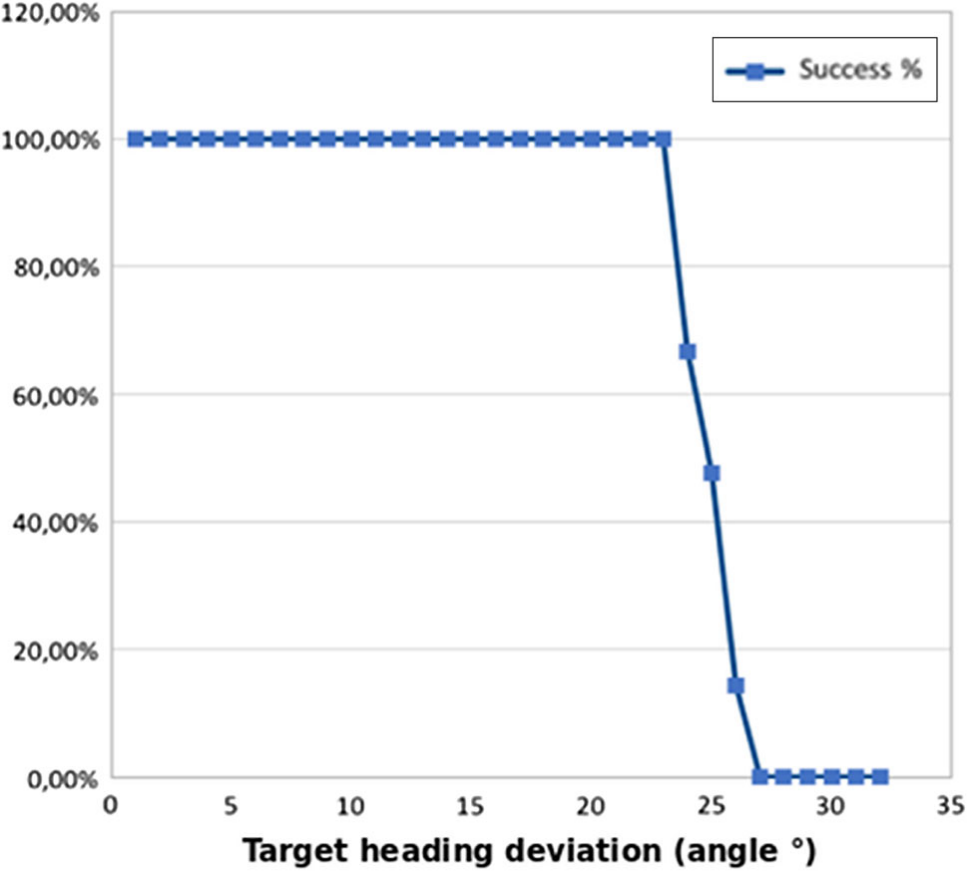
The graph shows the AHFT success rate with respect to an increasing deviation of the target heading from the coaxial start and target scenario

Following these results, an automatic parametrization method was developed to enable case-specific AFT efficiency optimization, as follows:

- Select the number of AFT levels (N) and AFT density $\rho$ according to hardware constraints.
- Compute the branch length value necessary to cover the search space through the N-level AFT growth based on the distance between a given start and target positions (which can be less than the predefined 100 mm used during the previous simulations).

This optimization method, applied to our test case, results in a 5-level AFT, with an AFT density equal to 17, which results in 1,508,597 tree branches. This resulting optimized AFT is used to demonstrate AHFT performance in subsequent sections. Three different tests were performed to assess the performance of the AHFT-based path planner. The first two focus on the robustness of the AHFT to changes in target pose and final heading, respectively, with no obstacles included in the search space. These tests explore the ability of the algorithm to respond to changes in the plan incurred due to, e.g., tissue deformation, deliquoration and pulsatile motion. The last test involves the simulation of a preoperative scenario, which enables the assessment of the AHFT within a complex network of realistic obstacles. Here, the use of our “reachable volume” computation (“AHFT algorithm implementation” section) to automatically support user selection of a viable entry point is also described. Such a tool is expected to simplify the planning process in complex neurosurgical scenarios, where the range of feasible paths is highly restricted.

### AHFT robustness evaluation

Here, we evaluate the ability of the AHFT to identify feasible paths for the following set of needle kinematic constraints: curvature continuity, maximum curvature ($k_{\max }$=$\frac{1}{70} \hbox {mm}^{-1}$) and needle length ($l=$ 100 mm). Starting with the trivial case of a straight line path between the entry and target pose, we then systematically alter both the position and the approach vector of the target pose to explore the bounds of the solution space. In doing so, we provide a visual and quantitative representation of needle performance, confirming correct execution within the solution space.

**Fig. 5:**
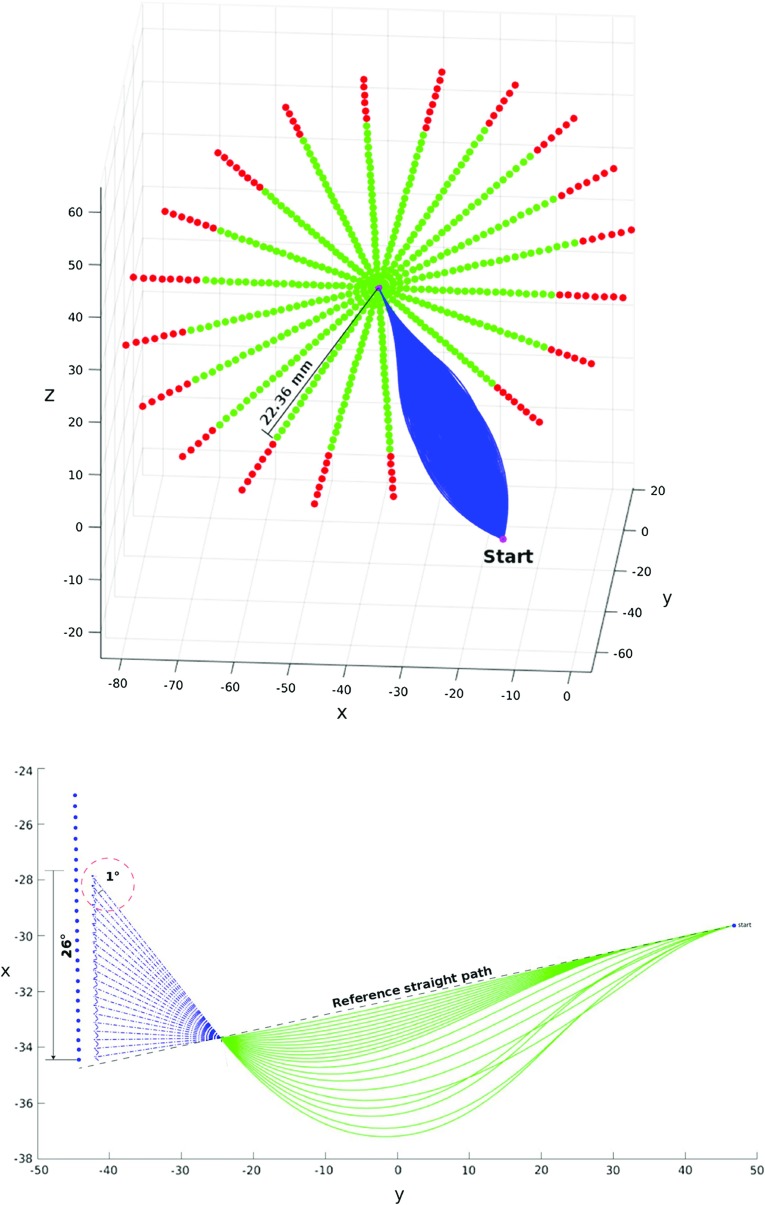
AHFT robustness evaluation for target approach vector: given a target position and an initial pose, the final approach vector is systematically oriented away from the trivial case until no solution is found. A red point corresponds to an approach vector for which no paths are found, while green points correspond to successful runs. At the bottom, the solutions corresponding to a single ray of the polar grid are displayed

*Target Orientation Sensitivity* To ascertain the sensitivity of the algorithm to target approach vector changes, a setup with coaxial start and end approach vectors is considered, then the target approach vectors are displaced with steps of one degree toward a certain direction until no solution is found. This is repeated for 21 displacement directions that constitute the rays of a polar grid where the angular spacing is equal to $\frac{\pi }{10}$. Each approach vector was then considered as the target approach vector for an AHFT run, maintaining the same target position and the same starting pose for all pairs within this test. Results are provided in Fig. 4, where the success rate for each displacement degree is calculated as the number of times at least a path is found with respect to the total number of directions evaluated. The green points identify those target approach vectors for which at least one path could be found, while red points correspond to those for which no paths were found (Fig. 5). From these results, solutions could be found for all target coordinates within 23$^{\circ }$ of the coaxial start and target scenario. The success rate, defined as the number of target points providing at least one AHFT solution, decreases to 66.66% for a deviation of 24$^{\circ }$, to 47.61% for 25$^{\circ }$ and finally to 14.29% for 26$^{\circ }$, which is the maximum deviation for which we can still find some solutions.

**Fig. 6:**
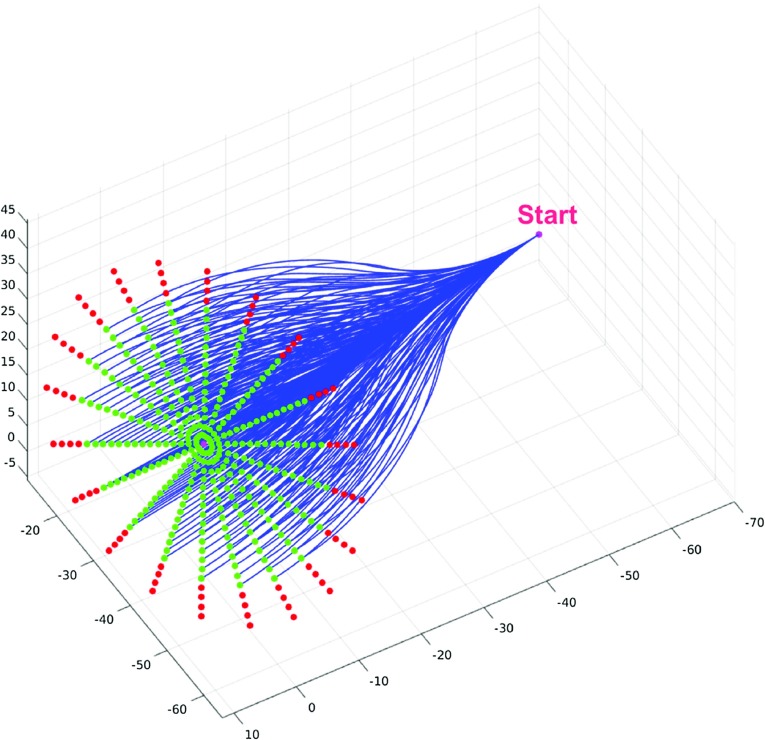
AHFT robustness evaluation for target placement: given a final approach vector and a fixed starting pose, the target position is systematically moved away until no solutions can be found. A red point indicates that no paths are found for the given target position, while a green point corresponds to a successful run

**Fig. 7:**
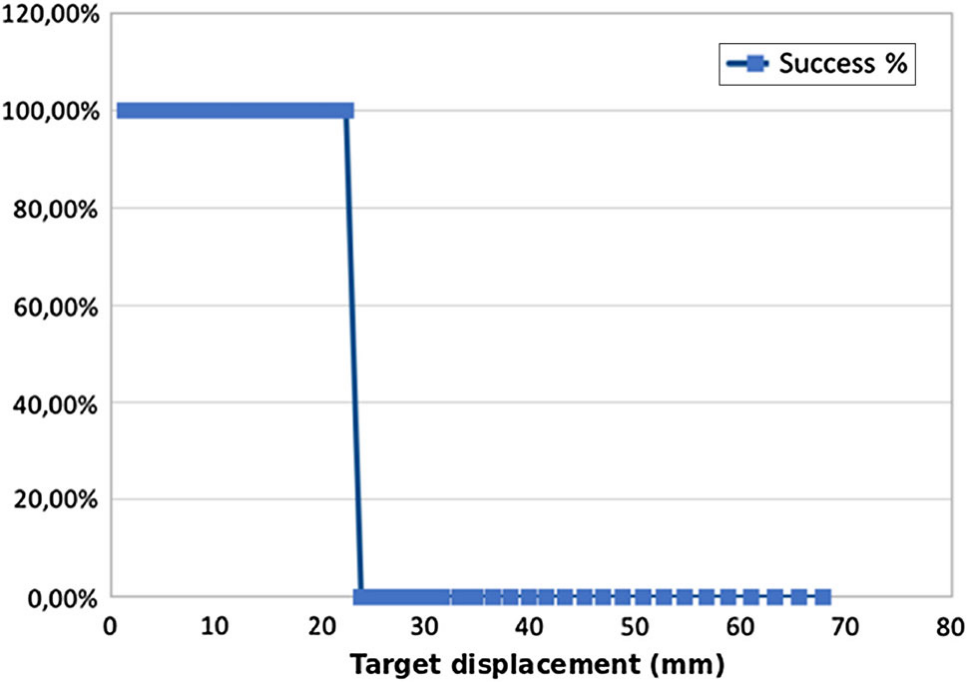
The graph shows the AHFT success rate with respect to an increasing displacement of the target away from the polar grid center

*Target Placement Sensitivity* To ascertain the sensitivity of the algorithm to target placement, the following setup was employed. Starting with the trivial case of a straight line path with coaxial start and end approach vectors, target coordinates were discretized within the same polar grid described in “AHFT robustness evaluation” section, maintaining both approach vectors constant. Each point within the grid was then considered as the target for an AHFT run, maintaining the same starting pose for all pairs within this test. Results are provided in Fig. 6, where green points identify those targets for which at least one path could be found, while red points correspond to those for which no paths were found. From these results (Fig. 7), it is evident that solutions could be found for all target coordinates within a 22.36 mm radius circle from the center. Once again, these results confirm the ability of the AHFT algorithm to identify suitable solutions within the feasible space.

**Fig. 8:**
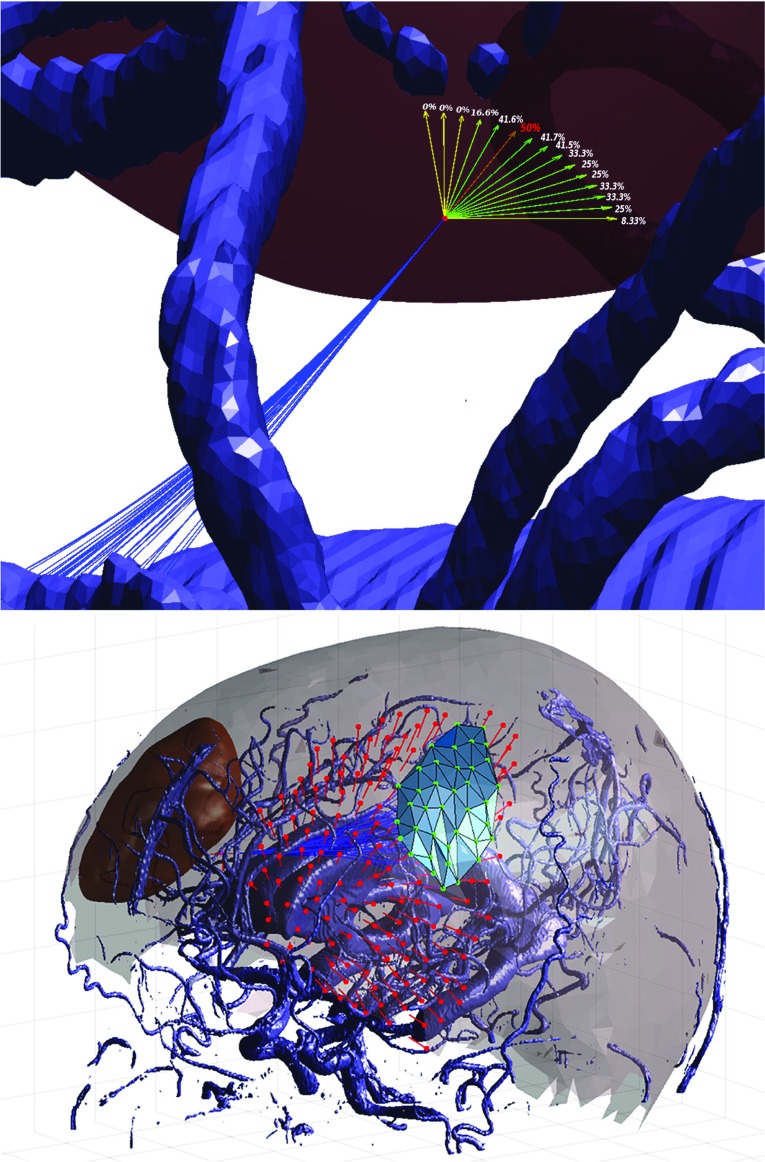
Preoperative scenario simulation: for each one of the displayed target poses laying near the tumor (violet), a rate has been calculated considering the number of feasible entry poses from which at least a path could be found with respect to the total skull mesh vertices. On the bottom, the best target pose (in terms of rate) is considered, and a skull region (light blue) is displayed to highlight the surface area where a burr-hole could be placed. Within this highlighted region, all feasible paths between each mesh vertex and the target are depicted in blue. Vertices in green are associated with obstacle-free paths, while red vertices are those for which an obstacle-free path could not be found

*Preoperative Path Planning Simulation* In a preoperative neurosurgical scenario, the operating surgeon would generally select a suitable entry point for the needle on the skull of the patient. This decision has to account for the desired target pose (i.e., the position and orientation of the needle at the point of application) and any cortical functional areas that must be avoided. Additionally, the approach angle at the start of the needle insertion process should be roughly perpendicular to the skull at the desired entry point in order to facilitate the creation of a suitable burr hole. Consequently, the selection of a feasible starting pose is not straightforward. To aid in this process, each vertex of a homogeneously distributed skull mesh is considered in turn (total number of vertices $=$ 1702), where the coordinates represent a possible entry point, and the associated vertex normal represents the corresponding desired start approach vector. All feasible entry points for a given target pose are then identified by executing the feasibility check for each pair, as per “AHFT algorithm implementation” section. The green dots within the light blue region are those for which the AHFT is able to find at least one trajectory, while the red dots are those for which no paths can be found because of obstacle collisions, which are not accounted for during the feasibility check. For illustrative purposes, a second mesh is produced out of the green successful vertices, in such a way as to highlight the skull region which would be suitable for a given target pose. Such a region is highlighted in light blue in Fig. 8, as an illustrative case, alongside a complete set of feasible paths (dark blue) for a representative target pose close to a tumor.

The skull mapping was performed for 15 different target poses. The average computation time for every start and target pose pair was 24.67 s, with a high standard deviation (25.76 s) because the path planner was run until the first solution was found, leading to different times depending on task complexity. The target pose success rate for all runs is shown in Fig. 8.

Each rate has been computed considering the number of feasible entry poses from which at least a path could be found with respect to the total corresponding amount of feasible points for the considered target pose. The fact that, for some target headings, few solutions could be found is mainly due to the density of the obstacle map and the complexity of the selected case in terms of start and target poses. These target poses are possibly those which a surgeon should exclude in favor of ones highlighted in the subregions, which would guarantee a better coverage of the feasible area on the patient skull (red dashed target pose in Fig. 8).

## Discussion

Starting with the trivial case of a straight line path between entry and target pose, systematically changing both the position and the approach vector of the target pose resulted in a set of results which confirm the ability of the AHFT algorithm to identify suitable candidate paths, if they exist. As the simulated setup explored increasingly challenging pose configurations (i.e., those lying at the edges of the solution space), more complex trajectories were required to intersect the target pose, implying a greater risk of failure. The AHFT algorithm is thus able to find at least one path for a given set of needle constraints, as long as the AFT sampling space lies within the “feasible volume” identified in “AHFT algorithm implementation” section. The AHFT is a sampling-based method which, because of its discrete nature, does not guarantee an optimum solution. However, the fractal tree structure provides a dense, invariant and organized exploration of the entire domain ensuring high robustness and success rate in path planning for highly constrained and complex environments. The top-ranked generated trajectories with respect to a specific cost function are those to be selected. The AHFT architecture also lead itself to full parallelization, unlocking the massive computational speedup capacity of the GPU, leading to a potential for intraoperative use. The differences in the experimental setup, ways to access the methods, software and hardware used make it difficult to compare the performance of path planning algorithms completely. Additionally, the very different constraints related to medical applications influence the complexity of the problem, making path planning algorithms very case-specific and even more difficult to assess. However, with respect to the preoperative simulation scenario, the proof of concept results described in “AHFT robustness evaluation” section demonstrate that the AHFT is able to identify a path planning solution if one can be found, and that the method can be used to identify a dense set of viable skull entry points within a preferable skull entry region, for a given target pose and set of needle constraints, automatically. Clinicians would still be free to select any other point within the highlighted skull region/s, as needed. The method, therefore, can offer an important tool to assist and facilitate planning in the most complex surgical scenarios. The surgeon can be assisted in identifying a suitable location for the burr hole, which is both clinically safe and feasible, with full control over both the entry and target needle poses.

## Conclusion

In this work, we proposed the Adaptive Hermite Fractal Tree, a novel parallelizable 3D path planning approach able to cope with the kinematic constraints of a steerable needle and predefined start and target poses. The performance of the algorithm to perturbations in the target position and approach vector were evaluated. Additionally, the AHFT was tested in a preoperative neurosurgical simulated environment, which demonstrates that multiple viable paths can be identified through a complex network of realistic obstacles. The method also enables easy identification of a suitable entry area on the patient skull for a given choice of target pose, which would be a useful tool for the surgeon. AHFTs can be applied to other fields where explicit control on entry and target poses, and a parallelizable architecture, are required. Future work will focus on parallelization of the AHFT algorithm. In particular, the OGH extensions departing from the AFT trajectories at different levels could be independently evaluated on the GPU. The voxel-based obstacle collision and the curvature check would then be performed in parallel in a similar way to the AFT method which covers the first part of the generated path. This would drastically reduce computation time and thus enable an online application of the method for intraoperative use.
